# The Inauguration of the Röntgen Society

**Published:** 1897-11-13

**Authors:** 


### THE INAUGURATION OF THE RÖNTGEN SOCIETY.

The inaugural meeting of the Röntgen Society at the St. Martin's Town Hall on Friday last was a very brilliant affair. The rooms were crammed with the "fair and brave." Not even the life-sized photograph of a lady, whose skeleton, softer tissues, and attire were all forthshadowed, deterred the throngs that waited patiently outside the dark rooms, awaiting their turn to study living anatomy in their own persons, or in those of their friends. From all sides came the whirr of motors, and the cracking of electric sparks, and the buzz of conversation, in which the learned few explained the marvels of the new science to the unlearned many. Excellent music was provided and lantern displays. The event of the evening, however, was Professor Silvanus Thompson's eloquent, instructive, and scholarly address. After sketching in a masterly manner the discovery of the new light, which made darkness visible, on November 8th, 1895, and the subsequent discovery that the rays acted on sensitised paper, and so photographed the objects they revealed, Professor Thompson alluded to the experimenters who began with photography, and finding sciagraphy anew, imagined themselves its original discoverers. Whereas, Professor Röntgen first found the sciagraphic and subsequently the photographic powers of the X rays. Professor Thompson discussed all sorts and conditions of screens and coils, tubes and apparatus. What was known and what was not known of the wonderful light that differed in so many ways from every other light. He plunged into theories as to what they really were, and what they were likely to do. He reviewed the problems yet to solve, the facilities provided for this purpose in the little university of Würtzburg and the absence in London of a college for physical science. The sections bearing upon surgery and medicine were granted the most important place in the address. Professor Thompson classed the X rays as the third important advance in modern surgery. First came that of anæsthetics, second antiseptics, and lastly the black art of sciagraphy. Professor Thompson's definition of the discovery as "fortunate but not fortuitous, because Herr Röntgen was looking for something, though he did not know what he was looking for," was very happy, and equally appropriate was the concluding sentence of his oration—"Nature never yet betrayed the heart that loved her."

